# Impact of Titanium Plate Fixation on Diacylglycerol and Growth Factor Levels in the Periosteum of the Mandible and Maxilla in Patients with Dentofacial Deformities After Jaw Osteotomies

**DOI:** 10.3390/ijms26052020

**Published:** 2025-02-26

**Authors:** Bożena Antonowicz, Jan Borys, Kamila Roszczyc-Owsiejczuk, Kamila Łukaszuk, Anna Zalewska, Agnieszka U. Błachnio-Zabielska

**Affiliations:** 1 Department of Dental Surgery, Medical University in Bialystok, 15-089 Białystok, Poland; 2 Department of Maxillofacial and Plastic Surgery, Medical University of Bialystok, 15-089 Białystok, Poland; jan.borys@umb.edu.pl (J.B.); kamila.lukaszuk@umb.edu.pl (K.Ł.); 3 Department of Hygiene, Epidemiology and Metabolic Disorders, Medical University of Bialystok, 15-089 Białystok, Poland; kamila.roszczyc-owsiejczuk@umb.edu.pl; 4 Department of Restorative Dentistry, Medical University of Bialystok, 15-089 Białystok, Poland; azlewska426@gmail.com

**Keywords:** titanium plates, growth factors, diacylglycerols, periosteum, maxilla, mandible

## Abstract

Titanium is widely recognized for its biocompatibility and utility in maxillofacial and orthopedic surgery; however, its influence on bone remodeling biomarkers remains underexplored. This study investigates the effects of uncoated titanium plates on both the growth factors and diacylglycerols (DAGs) in the periostea of the maxilla and mandible, as DAG signaling is an essential secondary messenger molecule involved in intracellular signaling connected to various growth factors. The study group comprised 20 patients undergoing bimaxillary osteotomies using miniplates and screws made of Ti6Al4V titanium, from whom bone fixations were removed, while the control group included 20 patients operated on for dentofacial deformities (before the insertion of titanium fixations). Diacylglycerol levels in the serum and periosteum were analyzed using tandem mass spectrometry coupled with ultra-high performance liquid chromatography. Growth factors in the periosteum were measured via ELISA with commercially available assay kits. Our findings demonstrate a significant reduction in growth factors, including IGF-1, PDGF, and FGF-23, alongside decreased total DAG levels, suggesting titanium plate stabilization may modulate bone remodeling dynamics. Notably, while overall DAG levels declined, specific DAG species such as C16:0/16:0 and C18:0/18:0 were elevated, whereas polyunsaturated DAGs showed reductions, indicating selective regulation of lipid signaling pathways. Correlation analyses highlighted complex interactions between growth factors and DAGs, with distinct regional differences observed in the mandibular and maxillary periostea. These alterations may result from chronic titanium exposure, potentially inducing a low-grade immune response or modifying the local biochemical environment. This study emphasizes the need for further research into the long-term effects of titanium implants, particularly their influence on lipid metabolism, growth factor dynamics, and bone healing.

## 1. Introduction

Fractures of the mandible and maxilla constitute the majority of fractures within the viscerocranium [1]. Traditionally, fractures in this area were primarily stabilized through intermaxillary fixation using stainless steel wires that immobilized the jaws by securing them together [2]. However, titanium plates have now become an integral part of contemporary surgical practices for mandibular and maxillary stabilization after bimaxillary osteotomies and trauma, offering improved fracture alignment and promoting effective bone healing [3,4]. Titanium, known for its high biocompatibility, strength, and corrosion resistance, provides substantial benefits in terms of stability and tissue integration, particularly in maxillofacial applications. However, titanium plates and screws are also essential in various other areas of maxillofacial surgery, extending beyond trauma management and reconstruction. Their applications include the treatment of facial fractures, such as fractures of the maxilla, mandible, zygomatic bones, nasal bones, orbital walls, and the cranial vault [5]. The use of titanium fixation ensures stability and proper healing of bone segments, which is critical for achieving optimal functional and aesthetic outcomes. In orthognathic surgery, titanium plates play a key role in stabilizing bone segments following osteotomies performed to correct dentofacial deformities. Their biocompatibility and mechanical properties allow for effective bone healing while minimizing complications. Similarly, in oncologic maxillofacial surgery, titanium plates are extensively used for the reconstruction of bone defects after tumor resections. They also play a vital role in securing both free and microvascularized bone grafts, facilitating the restoration of facial structures. Furthermore, titanium plates are widely utilized in implant-prosthetic surgery, particularly in bone augmentation procedures and the reconstruction of bone defects to support dental implants. Their application extends to the management of congenital and acquired facial deformities, where they provide stability for corrective bone procedures. In cases involving the temporomandibular joint (TMJ), custom titanium plates are utilized for reconstruction after joint resection or in the treatment of severe ankylosis [6]. Given their broad and versatile applications, titanium plates remain a fundamental component of maxillofacial surgery, contributing to the successful treatment of a wide range of conditions [7]. Their role in trauma, orthognathic, oncologic, implant-prosthetic, and TMJ surgery underscores their significance and adaptability in modern surgical practice. However, despite these advantages, the long-term impact of titanium on bone healing processes remains uncertain and requires further investigation, especially as it has been repeatedly shown that titanium particles can be released from implants, potentially initiating inflammation and associated oxidative stress [8]. It is also noteworthy that titanium plates, which remain in the body for extended periods, may influence not only immediate bone healing but also the ongoing physiological process of bone remodeling. The human skeletal system is a dynamic structure that constantly undergoes remodeling to adapt to mechanical demands, repair microdamage, and maintain mineral homeostasis. This process, driven by the balanced activity of osteoclasts and osteoblasts, is regulated by a complex interplay of growth factors and signaling molecules that control bone resorption and formation [9].

Diacylglycerol (DAG), a secondary messenger essential to the signaling pathways of various growth factors [10], plays a significant role in cellular processes supporting bone regeneration [11]. Investigating the effects of titanium plates on DAG-mediated pathways could provide valuable insights for optimizing bone regeneration therapies. As a critical secondary messenger in cellular signaling, DAG influences cell proliferation, differentiation, and migration. These lipid molecules, produced in the phosphatidylinositol pathway, activate protein kinase C (PKC), a key mediator in both physiological and pathological processes, including tissue repair and bone regeneration. During bone healing, DAG can modulate the actions of essential growth factors critical to bone tissue regeneration and remodeling [10,11]. Key growth factors such as fibroblast growth factor-23 (FGF-23), platelet-derived growth factor (PDGF), insulin-like growth factor-1 (IGF-1), and parathyroid hormone-related protein (PTHrP) play specialized roles in bone repair: FGF-23 regulates phosphate metabolism and mineralization [12,13]; PDGF promotes cell migration and proliferation [13,14]; IGF-1 enhances osteoblast differentiation and bone formation [15]; and PTHrP is involved in calcium regulation and osteoclast activation during bone resorption [16]. The interaction between PKC activation by DAG and these growth factors may significantly influence the bone remodeling process [10]. Additionally, DAG signaling can modulate osteoblast and osteoclast activity, both essential for bone remodeling [17]. To date, the effects of titanium plates on the regulation of DAG levels and growth factors involved in healing of the jaws after osteotomies, fracture repair, and/or bone remodeling have not been studied. Understanding the relationship between DAG signaling, growth factor activity, and the stabilization provided by titanium plates is crucial not only for advancing therapeutic strategies aimed at improving fracture healing in craniofacial regions but also for assessing whether these plates interfere with the physiological bone remodeling process.

## 2. Results

### 2.1. Clinical Characteristics of the Studied Groups

No significant differences were found between the blood parameters of the control and experimental groups, except for CRP protein concentration, which was significantly higher in the experimental group (*p* < 0.0001), and creatinine levels, which were significantly lower in the experimental group compared to the control group (*p* < 0.01). However, despite these differences, both CRP and creatinine levels remained within the normal range in both groups (Table 1).

### 2.2. Diacylglycerols

The total DAG level in the periosteum of the maxilla in the experimental group decreased from 31.54 pmol/mg to 20.56 pmol/mg tissue (*p* < 0.0001). When considering individual DAG species, the levels of C16:0/16:0, C18:0/18:0, and C18:0/18:1 increased in the experimental group compared to the control group, whereas the content of the other measured DAGs decreased compared to the control group, except for C18:0/20:4, whose level was similar in both groups. In the periosteum of the mandible, a decrease in the total DAG level was also observed in the experimental group compared to the control group. Regarding specific DAG species, an increase in the levels of C16:0/16:0 and C18:0/18:0 was noted in the experimental group, while the content of the remaining DAG species decreased, except for C14:0/14:0, whose level did not significantly differ between the groups. The results are presented in Table 2.

In the serum, an increase in total DAG concentration was observed in the experimental group compared to the control group. When examining individual diacylglycerols, no statistically significant differences were noted for C16:0/16:0 and C18:2/18:2 concentrations between the experimental and control groups. However, a significant decrease in the concentrations of C18:0/18:0 and C18:0/18:1 was observed in the experimental group compared to the control group, while the concentrations of the remaining DAGs increased in the experimental group in comparison to the control group (Table 3).

### 2.3. Growth Factors

**Figure 1:**
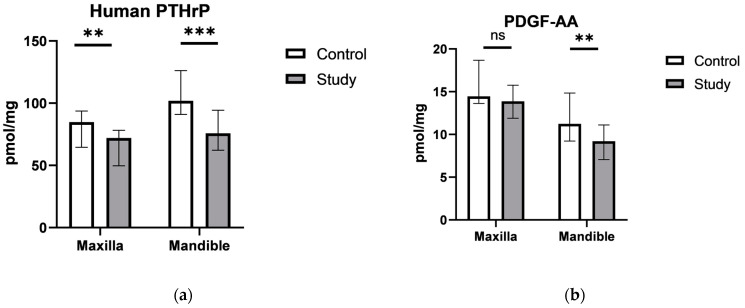

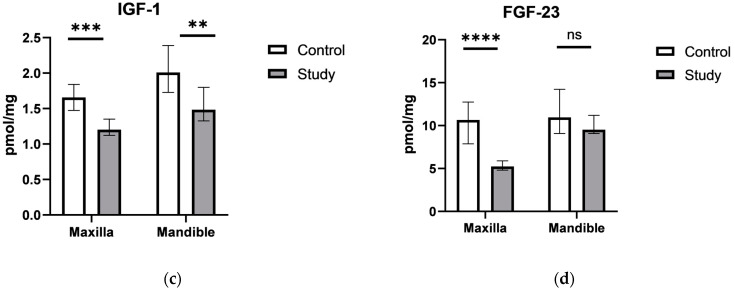
The concentration of: (**a**) Human parathyroid hormone-related protein (PTHrP); (**b**) Platelet-Derived Growth Factor AA (PDGF-AA); (**c**) Insulin-like Growth Factor 1 (IGF1); (**d**) Fibroblast Growth Factor 23 (FGF-23) in the periostea of the maxilla and mandible of the two study groups. Values are medians (interquartile range), and the differences between the groups were compared by the Mann–Whitney U test; ^ns^ *p*> 0.05; ** *p* ≤ 0.01, *** *p* ≤ 0.001, **** *p* ≤ 0.0001 indicate significant differences from the controls.

In the periosteum of the maxilla, a decrease in the levels of the human parathyroid hormone-like protein, IGF-1, and FGF-23 was observed compared to the control group, while in the periosteum of the mandible, a decrease in the human parathyroid hormone-like protein, PDGF, and IGF-1 was noted in comparison to the control group (Figure 1).

### 2.4. The Relationship Between DAG and Growth Factors

**Figure 2:**
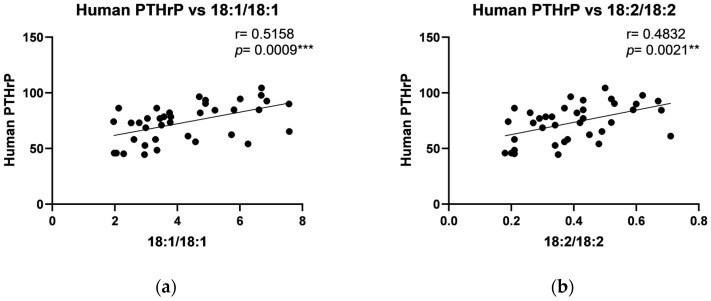

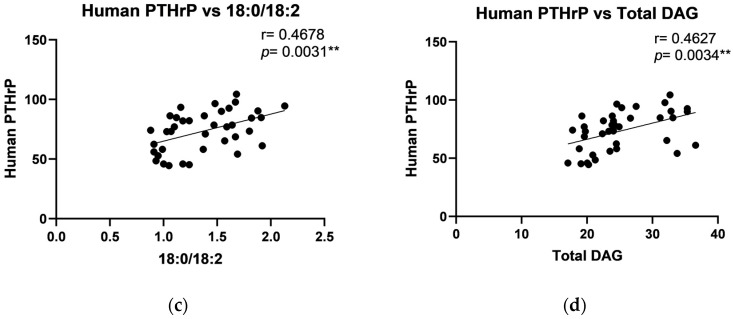
Correlations between the human parathyroid hormone-related protein (PTHrP) and particular diacylglycerols: (**a**) C18:1/18:1; (**b**) C18:2/18:2; (**c**) C18:0/18:2; (**d**) Total DAG in the periosteum of the maxilla. The data are presented as Pearson correlation coefficients; statistical significance: ** *p* ≤ 0.01, *** *p* ≤ 0.001. *p*-Value—probability, r—correlation coefficient.

**Figure 3:**
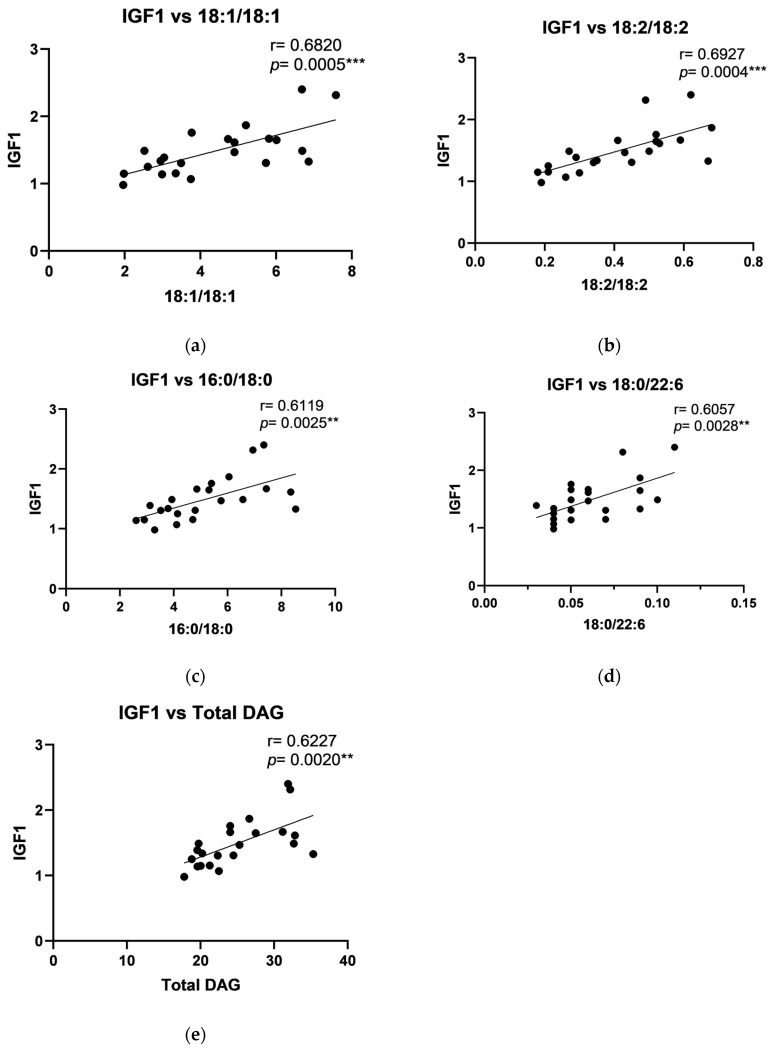
Correlations between Insulin-like Growth Factor 1 (IGF1) and particular diacylglycerols: (**a**) C18:1/18:1; (**b**) C18:2/18:2; (**c**) C16:0/18:0; (**d**) C18:0/22:6; (**e**) Total DAG in the periosteum of the maxilla. The data are presented as Pearson correlation coefficients; statistical significance: ** *p* ≤ 0.01, *** *p* ≤ 0.001. *p*-Value—probability, r—correlation coefficient.

**Figure 4:**
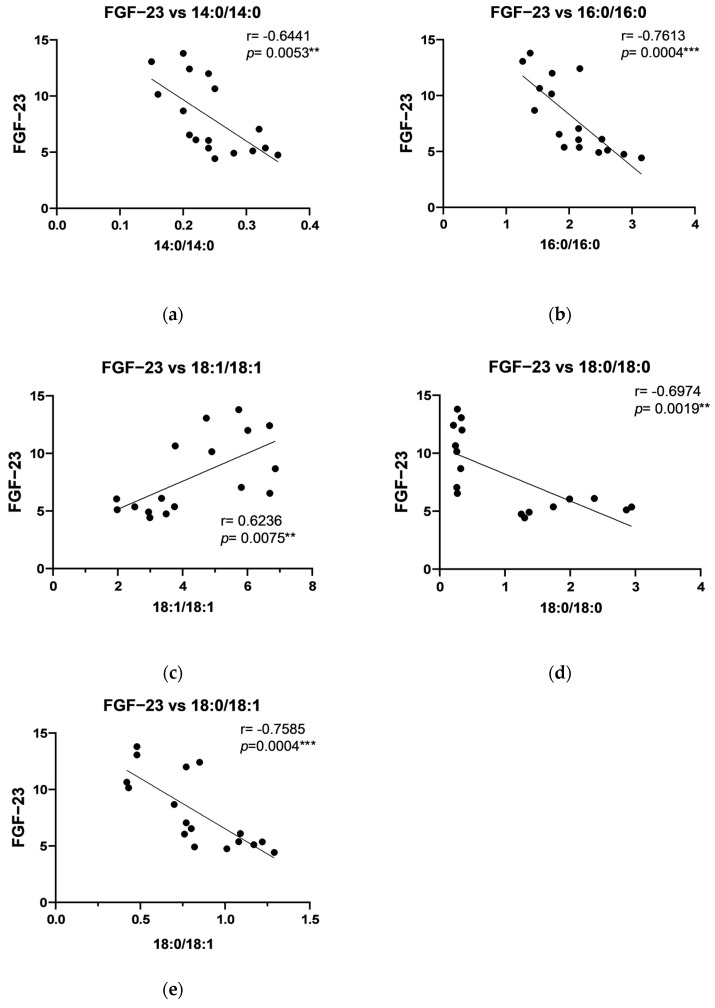
Correlations between Fibroblast Growth Factor 23 (FGF-23) and particular diacylglycerols: (**a**) C14:0/14:0; (**b**) C16:0/16:0; (**c**) C18:1/18:1; (**d**) C18:0/18:0; (**e**) C18:0/18:1 in the periosteum of the maxilla. The data are presented as Pearson correlation coefficients; statistical significance: ** *p* ≤ 0.01, *** *p* ≤ 0.001. *p*-Value—probability, r—correlation coefficient.

Correlation analysis showed positive correlations in the periosteum of the maxilla between human PTHrP and C18:1/18:1, C18:2/18:2, C18:0/18:2, and total DAG (Figure 2), and between IGF-1 level and C18:1/18:1, C18:2/18:2, C16:0/18:0, C18:0/22:6, and total DAG (Figure 3). Additionally, a strong negative correlation in the periosteum of the maxilla was noted between FGF-23 and C14:0/14:0, C16:0/16:0, C18:0/18:0, and C18:0/18:1 (Figure 4). Moreover, a positive, significant correlation was noted between FGF-23 and C18:1/18:1 (Figure 4).

**Figure 5:**
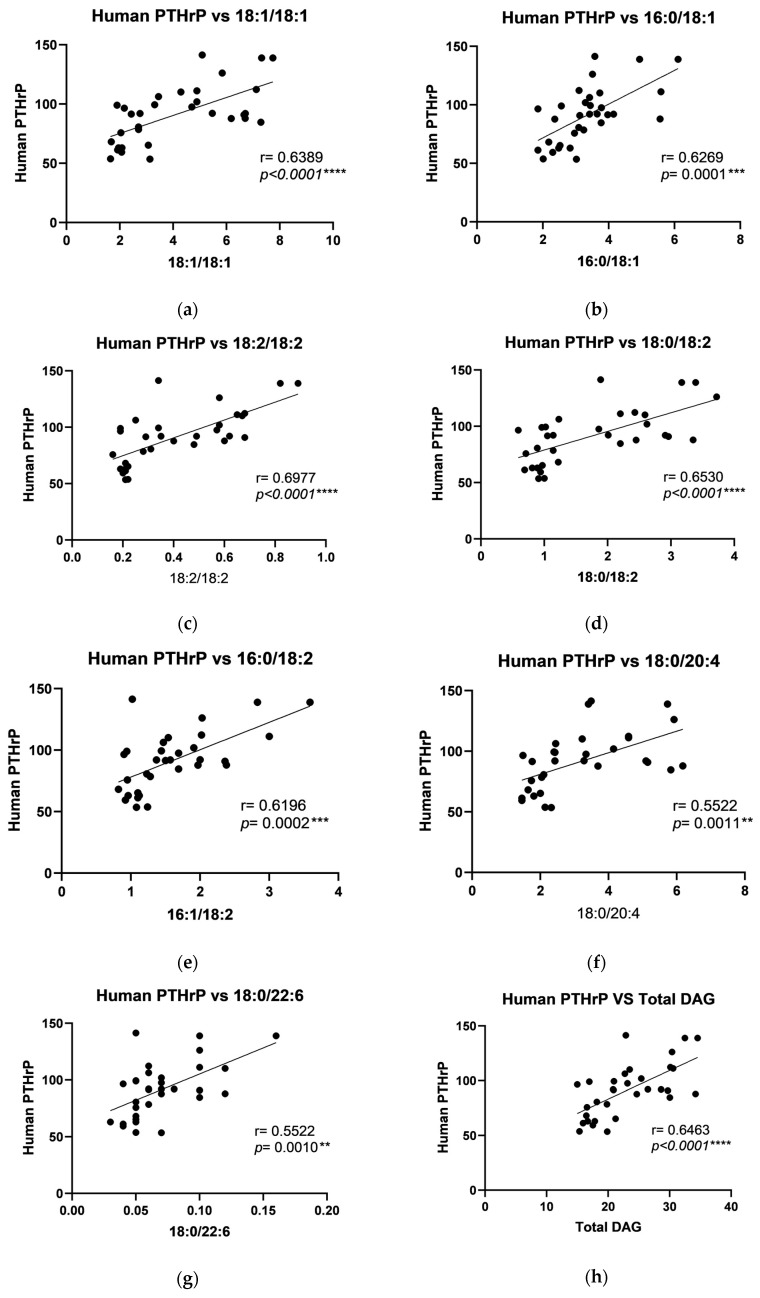
Correlations between the human parathyroid hormone-related protein (PTHrP) and particular diacylglycerols: (**a**) C18:1/18:1; (**b**) C16:0/ C18:1; (**c**) C18:2/18:2; (**d**) C16:0/C18:2; (**e**) C18:0/22:4; (**f**) C18:0/22:6; (**g**) C18:2/18:2; (**h**) Total DAG in the periosteum of the mandible. The data are presented as Pearson correlation coefficients; statistical significance: ** *p* ≤ 0.01, *** *p* ≤ 0.001, **** *p* ≤ 0.0001. *p*-Value—probability, r—correlation coefficient.

**Figure 6:**
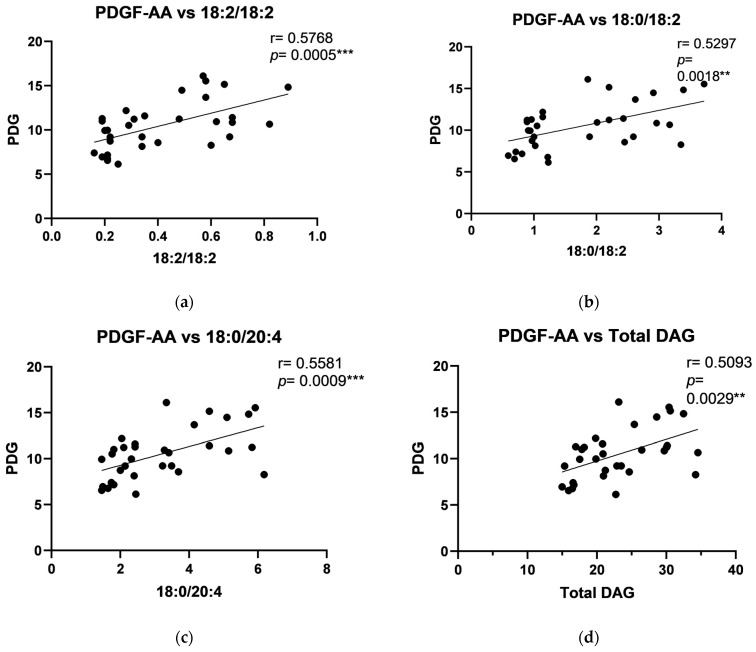
Correlations between Platelet-Derived Growth Factor AA (PDGF-AA) and particular diacylglycerols: (**a**) C18:2/18:2; (**b**) C18:0/ C18:2; (**c**) C18:0/20:4; (**d**) Total DAG in the periosteum of the mandible. The data are presented as Pearson correlation coefficients; statistical significance: ** *p* ≤ 0.01, *** *p* ≤ 0.001. *p*-Value—probability, r—correlation coefficient.

**Figure 7:**
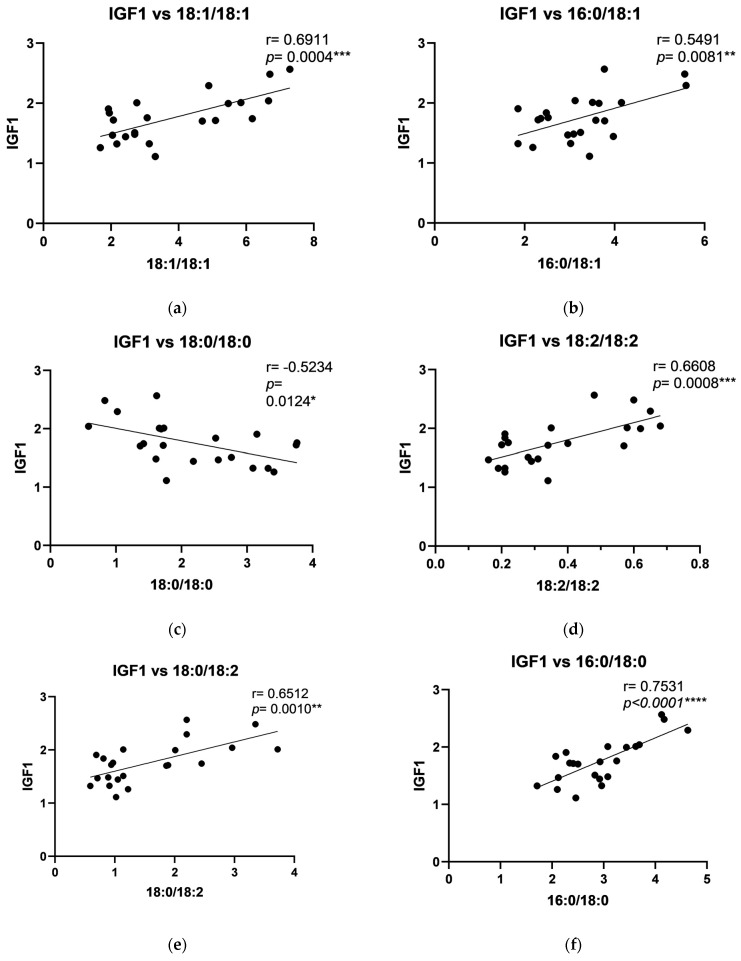

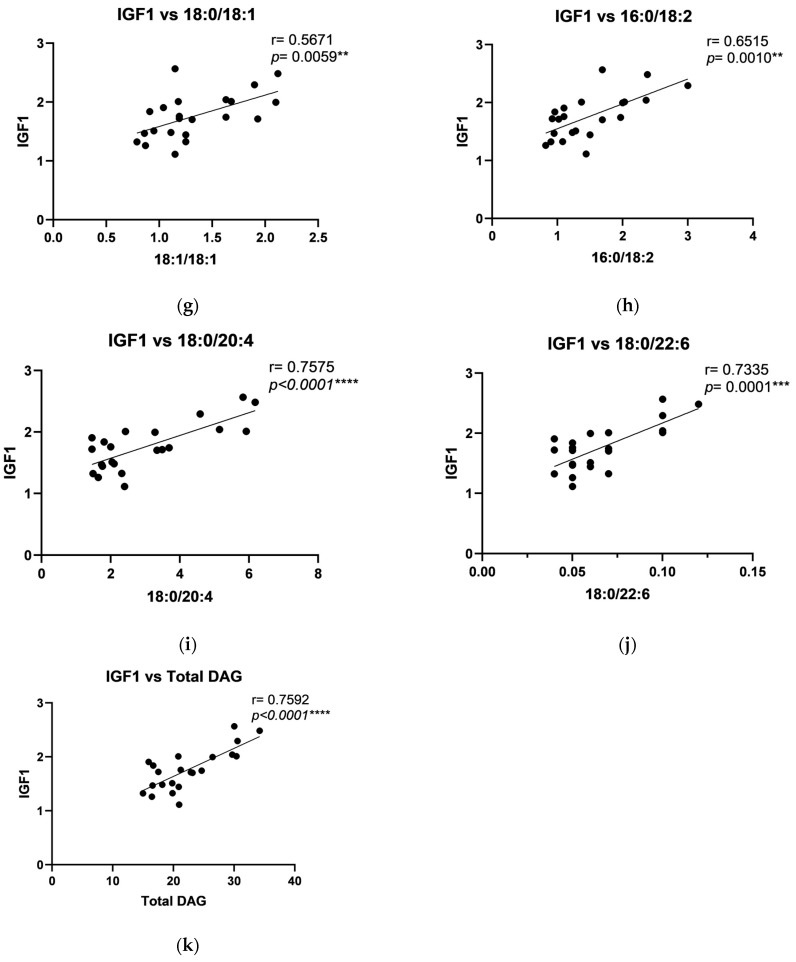
Correlations between Insulin-like Growth Factor 1 (IGF1) and particular diacylglycerols: (**a**) C18:1/18:1; (**b**) C16:0/C18:1; (**c**) C18:0/18:0; (**d**) C18:2/18:2; (**e**) C18:0/18:2; (**f**) C16:0/C18:0; (**g**) C18:0/18:1; (**h**) C16:0/C18:2; (**i**) C18:0/20:4; (**j**) C18:0/22:6; (**k**) Total DAG in the periosteum of the mandible. The data are presented as Pearson correlation coefficients; statistical significance: * *p* ≤ 0.05, ** *p* ≤ 0.01, *** *p* ≤ 0.001, **** *p* ≤ 0.0001. *p*-Value—probability, r—correlation coefficient.

In the periosteum of the mandible, positive correlations were observed between human PTHrP and C18:1/18:1, C16:0/18:1, C18:2/18:2, C18:0/18:2, C16:0/18:2, C18:0/20:4, C18:0/22:6, and total DAG levels (Figure 5). Positive correlations were also found between PDGF and C18:2/18:2, C18:0/18:2, C18:0/20:4, and total DAG in the periosteum of the mandible (Figure 6). Furthermore, a negative correlation between IGF-1 and C18:0/18:0, along with positive correlations with C18:1/18:1, C16:0/18:1, C18:2/18:2, C18:0/18:2, C16:0/18:0, C18:0/18:1, C16:0/18:2, C18:0/20:4, C18:0/22:6, and total DAG, was noted in the periosteum of the mandible (Figure 7).

## 3. Discussion

This study investigated the potential impact of titanium plates on the bone remodeling process markers, focusing on levels of growth factors associated with bone healing and the lipid signaling molecules—DAGs, which play a crucial role in cellular pathways linked to bone turnover. By examining changes in these biomarkers in the mandibular and maxillary periostea in patients with titanium implants, we aimed to determine if titanium plates influence remodeling dynamics, possibly by modulating DAG and the growth factors level.

Although growth factors are essential for bone healing and remodeling, the direct effect of titanium on the growth factor at the implant site remains complex and not fully understood. Titanium is a highly biocompatible, durable, and corrosion-resistant material widely used in medicine for manufacturing various types of implants. However, despite these favorable properties, numerous studies indicate that titanium implants are not entirely free from complications [18]. Studies show that titanium particles and ions may be released into surrounding tissues, during implantation or with long-term use, primarily due to mechanical wear, friction, and corrosion, especially under high-load conditions common in dental and orthopedic applications [19,20]. Released titanium ions and particles may initiate local inflammation or immune responses, a condition referred to as “metallosis”. Although titanium is biologically inert and generally well tolerated because of the TiO_2_ layer on their surface, the presence of titanium particles can activate macrophages and other immune cells, potentially triggering inflammation around the implant. In some cases, this inflammatory response may lead to bone resorption or reduce implant stability, especially in susceptible patients [21]. The migration of titanium particles into peri-implant tissues has been also linked to increased oxidative stress and local inflammatory responses, which may negatively affect bone-implant integration and overall implant longevity [22,23]. This inflammatory cascade involves macrophage activation, resulting in the release of pro-inflammatory cytokines and reactive oxygen species (ROSs) [24,25], which further amplifies oxidative stress in the surrounding tissues [26]. The elevated oxidative environment may impair osteoblast and fibroblast activity, both essential for successful osseointegration and the health of peri-implant soft tissues [27]. Emerging evidence further suggests that chronic exposure to titanium ions and nanoparticles may disrupt cellular homeostasis, adversely impacting the health of peri-implant tissues and highlighting a need for continued investigation into the long-term effects of titanium in medical implants [28]. However, data on the induction of oxidative stress by titanium are inconclusive. Some studies suggest that titanium may actually reduce oxidative stress [29]. The effect of titanium plates on other health aspects remains even less understood. To the best of our knowledge, the impact of titanium on DAG levels and growth factors in the periostea of the maxilla and mandible, which may also be associated with oxidative stress, remains largely unexplored. Research in this area is of particular importance, as stabilization plates are typically left at bone fusion sites for extended durations, raising questions about whether such implants may disrupt physiological remodeling by influencing growth factors and DAG levels.

Recent advancements in surface treatments, including anodization and bioactive coatings, have been designed to minimize corrosion and wear, thereby reducing the risk of titanium particles and ions releasing into adjacent tissues [30,31]. Coatings infused with growth factors, such as bone morphogenetic proteins (BMPs), IGF, and PDGF, aim to facilitate natural healing processes by delivering bioactive molecules that stimulate osteogenesis and angiogenesis, essential for efficient bone remodeling. By incorporating these growth factors directly onto titanium surfaces, the coatings create a localized microenvironment that encourages recruitment, proliferation, and differentiation of bone cells precisely at the fracture site. This biofunctionalization not only enhances osseointegration but also promotes faster and more consistent bone remodeling, potentially reducing overall healing time and minimizing complications associated with implant integration [32]. Such technological progress underscores the significant role of growth factors in orthopedic and trauma surgery, where optimized healing is paramount for successful clinical outcomes. Despite the importance of growth factors in regenerative and remodeling processes, the direct influence of titanium plates on the levels of these factors in surrounding tissues has not yet been studied.

Our data revealed a decline in growth factors, such as the human parathyroid hormone-like protein, IGF-1, and FGF-23, in the maxillary periosteum, with similar reductions in PDGF and IGF-1 in the mandibular periosteum. Moreover, our study indicated a significant reduction in total DAG levels in both maxillary and mandibular periostea of the study group, which suggests that the stabilization of bone segments with titanium plates may influence lipid signaling pathways, potentially affecting bone healing and remodeling. Specifically, the decrease in total DAG levels in the periosteum of maxilla highlights a notable shift in lipid composition. Interestingly, individual DAG species, such as C16:0/16:0, C18:0/18:0, and C18:0/18:1, increased in the maxillary periosteum of the experimental group, while others, including C18:1/18:1, C16:0/18:1, and C18:2/18:2, decreased, indicating a selective regulation of specific lipid molecules in response to titanium stabilization. These findings were similar to those observed in the mandible, where an increase in C16:0/16:0 and C18:0/18:0 DAG species contrasted with a decrease in C18:1/18:1 and other polyunsaturated DAGs.

The observed decrease in levels of IGF-1, PDGF, FGF-23, PTHrP, and total DAG following the implantation of titanium plates stands in contrast to the commonly held belief that titanium implants create a favorable environment for bone healing by supporting localized growth factor activity. It is important to note, however, that this expectation largely arises from studies conducted with titanium implants biofunctionalized with growth factors or other bioactive coatings. In contrast, the titanium plates used in this study were not coated with any such bioactive agents. This distinction may explain the reduced levels of growth factors and DAG observed, as the direct influence of uncoated titanium implants on growth factor activity has not yet been thoroughly investigated [30,32]. Moreover, the decrease in growth factor levels showed in our study suggest a response to long-term implant presence. Chronic titanium exposure could initiate a low-grade immune reaction or alter the local biochemical environment, potentially downregulating growth factor production over time. Data indicate that titanium particles may negatively affect the osteoblast function, inhibiting bone formation [33,34]; these results highlight a nuanced interaction that while short-term implantations enhance growth factor activity, longer exposure could suppress these factors as the body adapts to or compensates for the presence of the implant. Further research could elucidate whether this effect is due to physical wear, titanium ions, or an immune-modulatory response in long-term titanium plate usage.

Correlation analyses further underscored complex interactions between DAG species and growth factors, with positive correlations observed between human parathyroid hormone-like protein and specific DAGs (C18:1/18:1, C16:0/18:1, C18:2/18:2, C18:0/18:2, C16:0/18:2, C18:0/20:4, C18:0/22:6, and total DAG levels) and IGF-1 with C18:1/18:1 and other polyunsaturated DAGs in the periosteum of maxilla. In the mandibular periosteum, positive correlations were found between growth factors and both total DAG and specific DAG species, such as C18:2/18:2 and C18:0/20:4. The observed negative correlations between FGF-23 and certain DAGs (like C14:0/14:0 and C16:0/16:0) align with its role in phosphate metabolism and bone mineralization. The observed positive correlations between specific DAGs and growth factors, such as PTHrP and IGF-1, suggest that lipid signaling molecules may play a regulatory role in bone remodeling in response to titanium stabilization. Additionally, the correlation patterns in the periosteum of mandible, where PDGF levels showed positive associations with specific DAGs, further support the idea that local tissue-specific regulatory mechanisms influence bone turnover differently across skeletal regions. The differences between the maxilla and mandible indicate that local factors, such as mechanical loading, vascularization, or metabolic activity, may modulate these interactions. Furthermore, although titanium is known for its biocompatibility, long-term exposure may elicit subtle immune responses or oxidative stress, leading to alterations in local growth factor levels and lipid metabolism. Future studies utilizing advanced analytical techniques, such as metabolomic profiling and in vivo tracking of titanium particle release, could provide deeper insights into these mechanisms.

## 4. Materials and Methods

### 4.1. Ethics Approval and Consent

Patients in both, study and control, groups received comprehensive information regarding the study’s purpose, procedures for material collection (blood and periosteum), and potential risks. Written informed consent was obtained from each participant. The study protocol received approval from the Bioethics Committee of the Medical University of Bialystok (Approval No. APK.002.72.2023).

### 4.2. Patient Selection

A total of 40 patients diagnosed with Class III skeletal deformities (characterized by maxillary undergrowth and/or mandibular overgrowth) and undergoing bimaxillary osteotomy were recruited. All patients were treated at the Department of Maxillofacial and Plastic Surgery, Medical University of Bialystok, Poland, between 19 January 2023 and 30 November 2024.

The study group included 20 patients (12 females and 8 males, aged 20–28 years) who underwent bimaxillary osteotomies of the maxilla and mandible, followed by fixation with Ti6Al4V titanium alloy miniplates and miniscrews (ChM Lewickie Sp. z o.o., Lewickie, Poland). Four, 4-hole miniplates with four screws per plate were used bilaterally for fixation. Approximately 11 months postoperatively, following radiographic confirmation of bone union, the miniplates and screws were removed under general anesthesia. Radiological assessments included posterior–anterior (PA) projection teleroentgenograms and lateral cephalometry (Cranex 3D, Soredex).

The control group included 20 patients (14 females and 6 males, aged 21–30 years) who similarly underwent corrective surgery for dentofacial deformities (before insertion titanium fixations). All patients were generally healthy, with no history of systemic, inflammatory, or metabolic diseases, and were free from smoking, alcohol, or drug use. Exclusion criteria included any history of local inflammatory conditions, previous titanium implants, or inadequate oral hygiene.

### 4.3. Sample Collection

Blood samples were collected from fasting patients in the morning prior to surgery. A 10 mL sample from the antecubital vein was placed in anticoagulant-free tubes (Serum CAT, S-Monovette, Sarstedt, Germany) and centrifuged at 3000 rpm at 4 °C for 15 min (EBA 200, Hettich Zentrifugen, Tuttlingen, Germany). The serum was subsequently stored at −80 °C for biochemical analyses, including blood counts, coagulation profiles, and measurements of electrolyte, glucose, urea, AST, ALT, and CRP levels.

In the study group, periosteum samples (3 mm × 7 mm, 1 mm thick) adjacent to titanium plates on the maxilla and mandible were collected during fixation removal surgery. For the control group, similar periosteum samples were harvested from the maxilla and mandible during the initial osteotomy surgery, prior to miniplate insertion. All periosteum samples were obtained from areas that are routinely excised during these procedures.

### 4.4. Blood Analyses

Blood morphology was analyzed using specific methodologies to ensure accuracy and reliability across various parameters. White blood cell (WBC) counts were assessed through fluorescent flow cytometry, while red blood cells (RBCs), hematocrit (HCT), mean corpuscular volume (MCV), mean corpuscular hemoglobin concentration (MCHC), and platelets (PLTs) were measured using the conductometric method. Hemoglobin (HGB) levels were determined via spectrophotometry, with all these analyses performed on the SYSMEX XN 1500 analyzer. Platelets, prothrombin time (PT), and activated partial thromboplastin time (APTT) were measured using the STAR MAX2 device, which utilized a viscosity-based detection system. Additionally, electrolytes, C-reactive protein (CRP), cholesterol, and liver enzymes were measured on the Alinity platform using a chemiluminescent detection method.

### 4.5. Diacylglicerols Measurements

The level of DAG was measured by ultra-high performance liquid chromatography coupled with tandem mass spectrometry) (UHPLC/MS/MS) according to Blachnio-Zabielska et al. [35] with minor modifications. To each sample (tissue homogenates or plasma), 50 μL of the deuterated internal standard mix (Deuterated DAG Mixture I and Mixture II—Avanti Polar Lipids, Alabaster, AL, USA) as well as 2 mL of an extraction solution (isopropanol–water–ethyl acetate, 30:10:60; *v*:*v*:*v*) were added. The samples were then vortexed, sonicated, and centrifuged at 4000 rpm, 4 °C for 10 min. (Sorvall Legend RT, Thermo, Langenselbold, Germany). The supernatants were transferred to new vials, and the pellets were re-extracted. The supernatants were combined with the previous ones and evaporated under nitrogen. The dried samples were suspended in LC Solvent B (2 mM Ammonium formate, 0.1% formic acid in methanol) for UHPLC/MS/MS analysis. DAGs were separated using a reverse-phase Zorbax SB-C8 column (2.1 × 150 mm) in a binary gradient with Solvent A containing 1 mM ammonium formate and 0.1% formic acid in water and Solvent B containing 2 mM ammonium formate and 0.1% formic acid in methanol. The analysis was performed using a triple quadrupole mass spectrometer (Sciex QTRAP 6500 + AB Sciex, Darmstadt, Germany) against standard curves prepared for each compound.

### 4.6. Growth Factors Measurements

The levels of growth factors (human parathyroid hormone-like protein, PDGF, IGF-1, and FGF-23) (antibodies.com, St. Louis, MO, USA) in the periosteum were measured using commercially available kits, following the manufacturers’ protocols.

### 4.7. Statistical Analysis

The results were presented in the form of medians and interquartile ranges, specifically the 25th to 75th percentiles (IQR). Differences between the study and control groups were compared by means of the Mann–Whitney U test, and the relationship between variables was examined by means of Pearson correlation. A statistically significant threshold of *p* < 0.05 was applied. We conducted the statistical analysis by means of Prism 9.3.1 software made by GraphPad.

## 5. Conclusions

These findings expand on the existing literature linking DAG composition to bone metabolism, particularly its role in modulating anabolic signals essential for maintaining bone mass. The data indicate that DAG metabolism and the growth factor are closely interconnected, and their modulation through titanium plate stabilization could play a critical role in bone remodeling processes following maxillofacial surgery.

## Figures and Tables

**Table 1:** Blood chemistry test results (from patients before platelet removal surgery).

	Control Me (Q1–Q3)	Study Me (Q1–Q3)
WBC (×10^3^/µL)	6.5 (5.4–7.5)	5.9 (5.2–6.5) ^ns^
RBC (×10^6^/µL)	4.5 (4.2–5.0)	4.8 (4.5–5.2) ^ns^
HGB (g/dL)	15.7 (14.8–16.2)	14.8 (14.5–15.7) ^ns^
HCT (%)	47.4 (43.8–50.0)	45.1 (41.6–48.8) ^ns^
MCV (fL)	85.4 (82.3–89.9)	83.1 (81.3–88.6) ^ns^
MCHC (g/dL)	35.0 (34.0–36.1)	34.4 (32.7–35.2) ^ns^
PLT (×10^3^/µL)	272 (233–307)	239 (194–259) ^ns^
PT (s)	13 (12–14)	14 (12–14) ^ns^
APTT (s)	27.2 (26.3–29.6)	28.2 (26.6–30.4) ^ns^
INR (-)	1.00 (0.91–1.08)	0.98 (0.90–1.06) ^ns^
Na^+^ (mmol/L)	139 (136–142)	138 (136–140) ^ns^
K^+^ (mmol/L)	4.2 (3.7–4.8)	4.8 (3.7–4.9) ^ns^
Creatinine (mg/dL)	1.05 (0.89–1.12)	0.87 (0.78–1.04) **
Glucose (mg/dL)	90.1 (87.9–95.8)	86.5 (84.1–91.1) ^ns^
Urea (mg/dL)	28.33 (24.61–30.20)	25.74 (18.23–28.29) ^ns^
AST (U/I)	28 (25–32)	29 (26–32) ^ns^
ALT (U/I)	33 (29–37)	28 (22–37) ^ns^
CRP (mg/L)	1.4 (1.0–2.2)	2.8 (1.8–3.7) ****

Values are medians (interquartile range), and the differences between the groups were compared by the Mann–Whitney U test; ^ns^ *p* > 0.05; ** *p* ≤ 0.01, **** *p* ≤ 0.0001 indicate significant differences from the controls.

**Table 2:** The concentration of diacylglycerol in the periostea of the mandible and maxilla of the two study groups.

	Mandible		Maxilla	
	Control Me (Q1–Q3) (pmol/mg Tissue)	Control Me (Q1–Q3) (pmol/mg Tissue)	Control Me (Q1–Q3) (pmol/mg Tissue)	Study Me (Q1–Q3) (pmol/mg Tissue)
14:0/14:0	0.280 (0.230–0.320)	0.300 (0.250–0.365) ^ns^	0.225 (0.198–0.265)	0.285 (0.240–0.345) **
16:0/16:0	1.690 (1.370–1.850)	2.070 (1.865–2.650) ***	1.725 (1.373–2.030)	2.480 (2.113–2.825) ****
18:1/18:1	6.180 (4.890–7.120)	2.170 (1.935–2.915) ****	5.770 (4.723–6.683)	2.975 (2.340–3.418) ****
16:0/18:1	3.650 (3.290–4.940)	2.830 (2.240–3.330) ***	6.540 (5.670–8.438)	3.125 (2.640–3.768) ****
18:0/18:0	1.420 (1.020–1.690)	2.570 (2.030–3.250) ****	0.265 (0.210–0.330)	1.975 (1.383–2.430) ****
18:2/18:2	0.600 (0.490–0.680)	0.2100 (0.195–0.285) ****	0.510 (0.430–0.605)	0.295 (0.210–0.348) ****
18:0/18:2	2.590 (2.200–3.170)	0.960 (0.850–1.095) ****	1.640 (1.175–1.835)	1.140 (1.008–1.388) **
16:0/18:0	3.560 (2.930–4.120)	2.460 (2.110–3.020) ***	6.755 (5.378–7.593)	4.020 (3.163–4.458) ****
18:0/18:1	1.650 (1.340–1.930)	1.110 (0.890–1.200) ****	0.645 (0.480–0.763)	1.105 (0.935–1.305) ****
16:0/18:2	2.000 (1.690–2.380)	1.100 (0.9450–1.325) ****	2.595 (2.243–3.238)	1.380 (1.203–1.773) ****
18:0/20:4	4.590 (3.410–5.730)	2.000 (1.690–2.360) ****	2.720 (2.133–3.003)	2.285 (2.065–2.498) ^ns^
18:0/22:6	0.100 (0.070–0.100)	0.0500 (0.0450–0.060) ****	0.075 (0.058–0.093)	0.055 (0.0400–0.070) **
Total	29.69 (24.65–30.56)	17.83 (16.51–20.85) ****	31.54 (24.53–33.28)	20.56 (19.31–23.70) ****

Values are medians (interquartile range), and the differences between the groups were compared by the Mann–Whitney U test; ^ns^ *p* > 0.05; ** *p* ≤ 0.01, *** *p* ≤ 0.001, **** *p* ≤ 0.0001 indicate significant differences from the controls.

**Table 3:** The concentration of diacylglycerol in the sera of the two study groups.

	Control Me (Q1–Q3)	Study Me (Q1–Q3)
14:0/14:0	20.64 (18.08–23.57)	28.54 (20.46–31.96) **
16:0/16:0	372.0 (336.9–444.5)	388.8 (341.4–443.2) ^ns^
18:1/18:1	2155 (1915–2580)	3105 (2616–3291) ****
16:0/18:1	1825 (1458–2253)	2667 (2087–3003) ****
18:0/18:0	52.67 (39.60–58.43)	38.96 (33.48–42.69) ***
18:2/18:2	1079 (893.3–1364)	1103 (939.6–1343) ^ns^
18:0/18:2	117.4 (101.5–138.4)	177.3 (158.9–189.6) ****
16:0/18:0	1750 (1359–2063)	2322 (1945–2774) ***
18:0/18:1	156.7 (129.5–174.0)	126.2 (108.6–145.7)*
16:0/18:2	892.7 (728.2–1016)	1688 (1415–1861) ****
18:0/20:4	21.69 (18.87–24.42)	48.26 (38.43–58.64) ****
18:0/22:6	2.135 (1.748–3.083)	4.000 (3.488–4.435) ****
Total	8426 (7224–9695)	11922 (10036–13372) ****

Values are medians (interquartile range), and the differences between the groups were compared by the Mann–Whitney U test; ^ns^ *p* > 0.05; * *p* ≤ 0.05, ** *p* ≤ 0.01, *** *p* ≤ 0.001, **** *p* ≤ 0.0001 indicate significant differences from the controls.

## Data Availability

This article contains complete data used to support the findings of this study.
